# Hybridized Mechanical and Solar Energy-Driven Self-Powered Hydrogen Production

**DOI:** 10.1007/s40820-020-00422-4

**Published:** 2020-04-09

**Authors:** Xuelian Wei, Zhen Wen, Yina Liu, Ningning Zhai, Aimin Wei, Kun Feng, Guotao Yuan, Jun Zhong, Yinghuai Qiang, Xuhui Sun

**Affiliations:** 1 Jiangsu Province Engineering Laboratory of High Efficient Energy Storage Technology and Equipments, School of Materials Science and Engineering, China University of Mining and Technology, Xuzhou, 221116 People’s Republic of China; 2 Jiangsu Key Laboratory for Carbon-Based Functional Materials and Devices, Institute of Functional Nano and Soft Materials (FUNSOM), Soochow University, Suzhou, 215123 People’s Republic of China; 3 Department of Mathematical Sciences, Xi’an Jiaotong-Liverpool University, Suzhou, 215123 People’s Republic of China

**Keywords:** WO_3_/BiVO_4_ heterojunction, Photoelectrochemical hydrogen generation, Triboelectric nanogenerator, Mechanical energy, Solar energy

## Abstract

Photoelectrochemical hydrogen generation is a promising approach to address the environmental pollution and energy crisis. In this work, we present a hybridized mechanical and solar energy-driven self-powered hydrogen production system. A rotatory disc-shaped triboelectric nanogenerator was employed to harvest mechanical energy from water and functions as a sufficient external power source. WO_3_/BiVO_4_ heterojunction photoanode was synthesized in a PEC water-splitting cell to produce H_2_. After transformation and rectification, the peak current reaches 0.1 mA at the rotation speed of 60 rpm. In this case, the H_2_ evolution process only occurs with sunlight irradiation. When the rotation speed is over 130 rpm, the peak photocurrent and peak dark current have nearly equal value. Direct electrolysis of water is almost simultaneous with photoelectrocatalysis of water. It is worth noting that the hydrogen production rate increases to 5.45 and 7.27 μL min^−1^ without or with light illumination at 160 rpm. The corresponding energy conversion efficiency is calculated to be 2.43% and 2.59%, respectively. All the results demonstrate such a self-powered system can successfully achieve the PEC hydrogen generation, exhibiting promising possibility of energy conversion.

**Electronic supplementary material:**

The online version of this article (10.1007/s40820-020-00422-4) contains supplementary material, which is available to authorized users.

## Highlights

- A hybridized mechanical and solar energy-driven hydrogen production system was developed.
- A rotatory disc-shaped triboelectric nanogenerator (RD-TENG) enables to harvest mechanical energy from water flow and functions as a sufficient external power source.
- WO_3_/BiVO_4_ heterojunction is fabricated as photoanodes in the self-powered photoelectrochemical (PEC) cell, and the hydrogen production rate reaches to 7.27 μL min^−1^ under sunlight illumination with the energy conversion efficiency of 2.59%.

**Electronic supplementary material:**

The online version of this article (10.1007/s40820-020-00422-4) contains supplementary material, which is available to authorized users.

## Graphical abstract

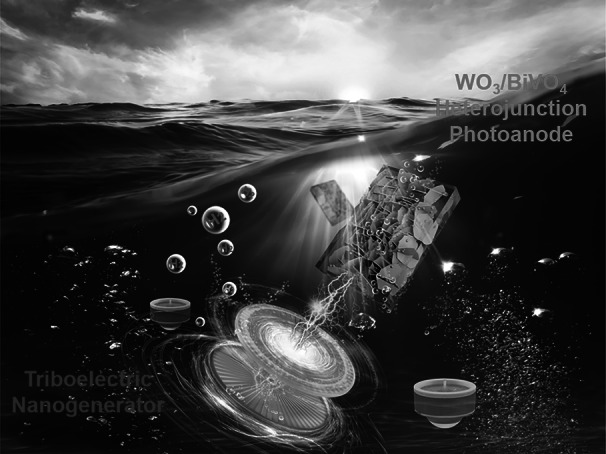

## Introduction

The serious energy crisis is an urgent global problem that mankind must turn to in the present and future. Hydrogen energy has been attracting increasing attention as a promising clean energy [1–3]. Through photoelectrochemical (PEC) water splitting, solar energy can be directly converted into hydrogen energy [4, 5]. However, in real applications, this process usually needs an external bias due to the improper band position of some semiconductor photocatalysts [6–11]. Morisaki et al. [12] constructed a TiO_2_-solar-cell hybrid electrode structure that undoubtedly provides an important development in PEC water splitting owing to the possibility of the application of an external bias generated by solar cells. After that, solar cells have been widely used in the field of PEC hydrogen production [13]. However, the considerable cost and complexity of the manufacture technology have impeded their commercial applications.

As an energy converter, triboelectric nanogenerator (TENG) can harvest various types of mechanical energies, such as human motion, wind energy, and hydropower [14–23]. The birth of TENG provides an approach as external bias for driving different electrochemical processes [24–26]. Accordingly, Tang et al. [27] developed a hybrid system constituted by coupling a TENG and a water-splitting unit and achieved fully self-powered water splitting for hydrogen generation. However, due to the peak output characteristics, the output of TENGs does not always keep at the peak value. At low voltage range, the electrolytic water-splitting process cannot happen at all, which greatly reduces the conversion efficiency. Soon after, another efficient strategy was proposed through PEC water splitting by simultaneously harvesting solar energy and mechanical energy [28–30]. Li et al. [31] developed a new type of TENG-PEC-based hybrid cell using TiO_2_ as photoanodes to obtain hydrogen. Nevertheless, limited by its wide band gap (3.2 eV), TiO_2_ can only absorb ultraviolet photons [32]. Before long, another efficient strategy was proposed through PEC water splitting by harvesting mechanical energy as an external bias to offset the band position of semiconductor photocatalysts [33]. Thus, exploring new photocatalytic materials as photoanodes towards this novel PEC hydrogen generation system attracts great attention.

In this work, a self-powered PEC hydrogen production system was successfully demonstrated to generate hydrogen. WO_3_/BiVO_4_ heterojunction nanostructure was prepared by water bath and electrodeposition method as photoanode in the PEC water-splitting cell to generate hydrogen. A rotatory disc-shaped TENG (RD-TENG) served as mechanical energy harvester based on the coupling effects of triboelectrification and electrostatic induction. After transformation and rectification, the generated electricity by RD-TENG acted as an external bias to achieve the overall PEC water splitting. The photocurrent output and dark current output under different rotation speeds were measured. Moreover, the hydrogen production rate under illumination had obvious increase compared to those of dark conditions. The detailed phenomenon and mechanism of the self-powered PEC hydrogen generation process have also been discussed. Finally, the whole system has been demonstrated to realize the PEC hydrogen generation.

## Experimental Methods

### Preparation of WO_3_ Photoanode

Fluorine-doped SnO_2_ glass (FTO, Nippon Sheet Glass, 14 O sq^−1^, Japan) was cut into blocks (5 × 3 × 0.2 cm^3^), and then dipped into acetone, ethanol, and deionized water for ultrasonic cleaning for 20 min, respectively. The precursor solution was obtained by the following two steps. Firstly, H_2_WO_4_ (0.6 g), (NH_4_)_2_C_2_O_4_ (0.28 g), HCl (37%) with 18 mL and H_2_O_2_ (37%) with 20 mL were added to 62 mL of deionized water; the second step is to add 60 mL of ethanol under strong agitation. The conductive surface of the previously cleaned FTO glass was dipped into the precursor solution in the water bath at 85 °C and kept for 3 h, after being naturally cooled to room temperature, washed with deionized water, and finally dried at 80 °C for 5 h. After placing the FTO glass in the autoclave, and finally annealed for 3 h with controlled temperature at 500 °C, the WO_3_ photoanode was obtained on the FTO substrate.

### Preparation of BiVO_4_ Photoanode

Bi(NO_3_)_3_·5H_2_O was mixed with 50 mL 0.4 M KI solution, and then HNO_3_ was added until the pH value was reduced to 1.7 to obtain a Bi(NO_3_)_3_ solution with 0.04 M. The above Bi(NO_3_)_3_ solution was mixed with 20 mL of anhydrous ethanol and 0.23 M p-benzoquinone by strongly stirring. An electrochemical workstation (CHI 660D) and a three-electrode cell was used for electrodeposition, where an Ag/AgCl electrode served as the reference electrode (RE), a Pt wire acted as the counter electrode (CE), and a cleaned FTO glass was regarded as the working electrode (WE). The deposition time was set to 10 min. Cathodic deposition was conducted at − 0.1 V versus Ag/AgCl potentiostatically at room temperature (RT), and finally the BiOI electrodes were obtained. A dimethyl sulfoxide (DMSO) solution including VO(acac)_2_ (0.2 M) was put on BiOI electrodes in 0.15–0.2 mL, then annealed for 2 h with 2 °C min^−1^ in the autoclave, and annealing temperature was set at 450 °C to obtain the BiVO_4_ electrode. To keep excess V_2_O_5_ of BiVO_4_ electrodes, the BiVO_4_ electrode in NaOH solution (1 M) was soaked for 30 min. The prepared BiVO_4_ electrode was washed using deionized water and dried at room temperature.

### Preparation of WO_3_/BiVO_4_ Photoanode

To prepare WO_3_/BiVO_4_ heterojunction photoanode, the fluorine-doped SnO_2_ (FTO) WE was replaced by the WO_3_ film, and the other experimental steps are the same as above.

### Fabrication of the RD-TENG

The stator: the matching acrylic sheets were cut as the supporting base board. The print circuit board (PCB) decorated with interdigital copper electrodes is attached to acrylic sheets. Then, a PTFE thin film is attached to the copper electrodes. Lastly, the rotator and the stator are mounted coaxially, and two conductors separated by an insulator are welded on the two copper electrodes.

The rotator: the laser cutter (Huitian Laser 4060) was used to cut the acrylic sheets (diameter, 184 mm; thickness, 3 mm) as the supporting base board. A PCB was deposited with an arrayed radially copper segment (central angle: 1.5° and thickness 70 μm). The PCB is made from stiff glass epoxy.

### Characterizations

A scanning electron microscope (SEM, FEI Quanta 200 F) and a high-resolution transmission electron microscopy (HRTEM, FEI/Philips Tecnai 12 Bio-TWIN) were used for the morphology characterization; meanwhile images of HRTEM and EDX spectroscopy were taken with a CM200 FEG transmission electron microscope. The structure was characterized by an X-ray diffraction (XRD, PANalytical, Empyrean) and an X-ray photoelectron Spectrometer (XPS, Kratos AXIS UltraDLD). The binding energies measured by XPS for each sample were calibrated on the basis of the C 1*s* peak (284.6 eV) [34]. UV–Vis spectra were performed by a Lambda 750 spectrophotometer. UPS spectra were measured on a He I (21.2 eV) gas discharge lamp. Hydrogen production rate was measured by a H_2_ collection tube (with a division of 20 μL): the Pt electrode was inserted into the H_2_ collection tube, where the H_2_ collection tube was fully filled with electrolytes, and then partly inserted into the electrolyser. The gas volume is recorded by the H_2_ collection tube during experiment from 100 to 160 rpm in darkness or under illumination. At last, the hydrogen production yield can be obtained by the ratio of gas volume and time.

### Electrical Measurement

A transformer (Taizhou Quanyi Electric Appliance Co., Ltd, EI24X13) was employed in the circuit for power management. A rotary motor (MODEL 86HSE8.5 N-B32) was used to drive the RD-TENG rotation, while a programmable electrometer (keithley-6514) was applied to test the *V*_oc_, *I*_sc_ and transfer-charge quantity (*Q*_tr_). Software based on LabVIEW platform, real-time data collection and analysis are realized.

### PEC Measurements

PEC tests were accomplished in a three-electrode cell with an Ag/AgCl electrode as the RE, a Pt wire as the CE, and the WE was served by the prepared photoanodes (back-side illumination). Photocurrent was measured in potassium phosphate (KH_2_PO_4_) buffer solution (0.5 M, pH = 7) with or without 1 M sodium sulphite (Na_2_SO_3_). All illuminated areas were 0.1 cm^2^. According to the following equation, the measured voltage was transformed into obtain a reversible hydrogen electrode (RHE) scale:

$$E_{\text{RHE}} = E_{\text{Ag/AgCl}}^{\theta } + E_{\text{Ag/AgCl}} + 0.059{\text{pH}}$$

where the $E_{\text{RHE}}$ is the calculated potential versus RHE, the $E_{\text{Ag/AgCl}}$ is the potential relative to the Ag/AgCl reference electrode, the pH is 7.1, and the $E_{\text{Ag/AgCl}}^{\theta }$ is equal to 0.1976 V at 25 °C. A light source (100 mW cm^−2^) was provided using the XD-300 xenon high-brightness cold light source with adjustable power under AM 1.5 G filter for the test. The scanning rate of the potential was 10 mV s^−1^ from 0.6 to 2.4 V versus RHE. Mott–Schottky plots were measured at a bias voltage from 0.4 to 0.1 V versus Ag/AgCl and a frequency of 1 kHz in the dark. We measured the electrochemical impedance spectra (EIS) at frequencies ranging from 10,000 to 0.1 Hz by applying 1.23 V versus RHE with amplitude of 10 mV in the light.

## Results and Discussion

**Fig. 1:**
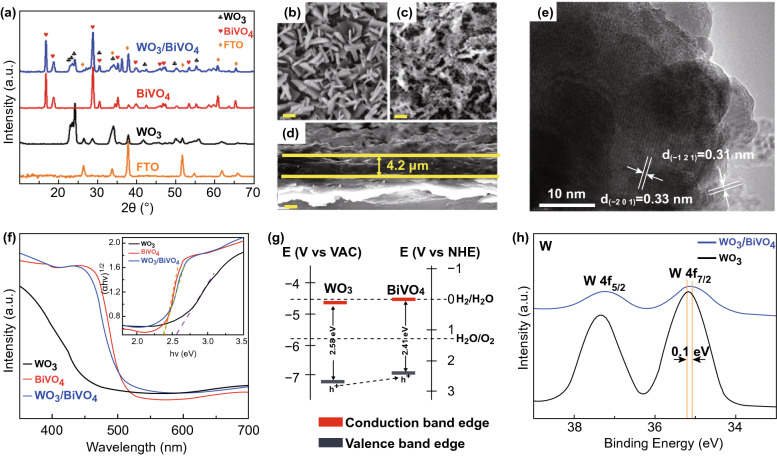
Characterizations of WO_3_/BiVO_4_ heterojunction photoanodes. a XRD spectra of the prepared photoanodes (WO_3_, BiVO_4_, and WO_3_/BiVO_4_). Top-view SEM images of **b** WO_3_, **c** WO_3_/BiVO_4_, and **d** cross-sectional SEM image of WO_3_/BiVO_4_ (scale bars, 2 μm). **e** HRTEM image of WO_3_/BiVO_4_ (scale bar, 10 nm). **f** UV–Vis spectra of the prepared materials. The inset shows Tauc’s plot analysis. **g** Band structure of WO_3_ and BiVO_4_. **h** High-resolution XPS curves of photoanodes at the W 4*f* edge

The characterizations of WO_3_/BiVO_4_ photoanodes prepared by water bath and followed by electrodeposition method are illustrated in Fig. 1. All the characteristic peaks in XRD patterns of the prepared photoanode belong to the WO_3_ (JCPDS No. 32-1395), BiVO_4_ (JCPDS No. 14-0688), and the FTO substrate (Fig. 1a), respectively. Compared with pristine WO_3_ nanoflake (Fig. 1b), the surface of WO_3_/BiVO_4_ seems much rougher (Fig. 1c), revealing that the WO_3_ surface was coated with the BiVO_4_ particles. The thickness of the WO_3_/BiVO_4_ photoanode layer is ~ 4.2 μm (Fig. 1d). Figure 1e shows the lattice fringe of *d* = 0.33 nm could be attributed to the (− 201) plane of WO_3_, and *d* = 0.31 nm is in agreement with the (− 121) plane of BiVO_4_, clearly indicating that BiVO_4_ nanoparticles were efficiently deposited on the surface of WO_3_ to form a heterojunction. The bandgap values of WO_3_, BiVO_4_, and WO_3_/BiVO_4_ were estimated using the UV–Vis absorption spectra, as shown in Fig. 1f, and the corresponding values are about 2.58, 2.41, and 2.38 eV, respectively [35]. It shows clearly that the light absorption range of WO_3_ is enlarged after coupling with BiVO_4_ and the visible-light absorption capacity is enhanced as well. From ultraviolet photoelectron spectroscopy (UPS) spectra of WO_3_ and BiVO_4_ (Fig. S1), we could calculate that the top of the valence band (VB) for these two materials is about − 7.2 and − 6.9 eV (relative to the level of vacuum), respectively (Supporting Note S1). Therefore, the band structure of WO_3_ and BiVO_4_ could be calculated using the UV–Vis absorption spectra and the UPS results (Fig. 1g), where 0 V in RHE equals to − 4.5 V in VAC. The electrode potential of the VB of the BiVO_4_ is significantly higher than that of the WO_3_, which is beneficial for the transfer of the photo-generated holes, thus reducing the recombination of the photo-generated electron–hole pairs, and ultimately the performance can thus be boosted by the heterostructure of WO_3_/BiVO_4_ [36–38]. The XPS spectra of WO_3_ and WO_3_/BiVO_4_ photoanodes at the W 4*f* edge show that the two obvious peaks of 35.2 and 37.3 eV in both samples could be regarded as W 4*f*_7/2_ and W 4*f*_5/2_ of W^6+^, respectively (Fig. 1f) [39, 40]. There is 0.1 eV offset here, which may be due to the formation of WO_3_/BiVO_4_ heterojunction [41]. The detailed XPS analysis of the WO_3_, BiVO_4_, and WO_3_/BiVO_4_ photoanodes is shown in Fig. S2.

**Fig. 2:**
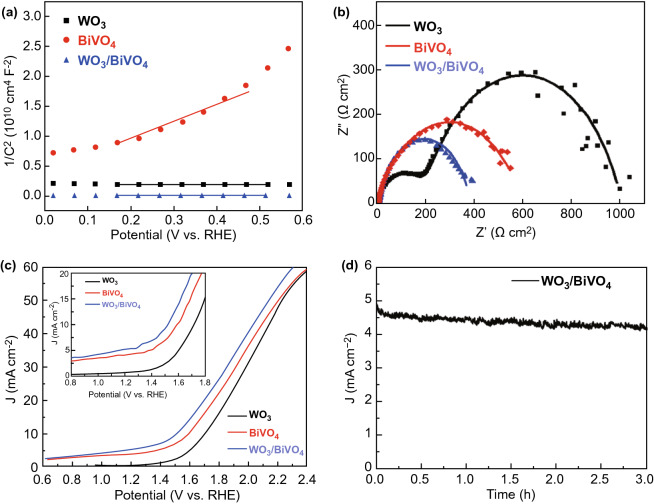
Photoelectrochemical performances of WO_3_/BiVO_4_ photoanodes. a Mott–Schottky plots and **b** EIS spectra of the photoanodes at 1.23 V versus RHE. **c** *J*–*V* curves of the photoelectrodes measured in phosphate buffer (0.5 M, pH = 7) including Na_2_SO_3_ (1 M) as hole scavenger. The inset exhibits the enlarged image of the potential (0.8–1.8 V vs. RHE). **d** Photochemical stability of the WO_3_/BiVO_4_ (1.23 V vs. RHE)

The photoelectrochemical performances of WO_3_/BiVO_4_ photoanodes are demonstrated using a three-electrode cell, as shown in Fig. 2. Figure 2a exhibits the Mott–Schottky plots of the photoanodes, and the charge carrier densities of WO_3_/BiVO_4_ and BiVO_4_ can be calculated as 6.81 × 10^23^ and 7.30 × 10^19^ cm^−3^, respectively, by the slope of the corresponding curves in figure (Supporting Note S2) [42]. Compared with the individual BiVO_4_, the WO_3_/BiVO_4_ heterojunction photoanode has the higher carrier density, which is beneficial for improving the performance to some extent. Furthermore, EIS spectra of WO_3_, BiVO_4_, and WO_3_/BiVO_4_ photoanodes were measured under simulated solar light illumination (Fig. 2b). It is discovered that the WO_3_/BiVO_4_ heterojunction photoanode has the minimum arc diameter, compared with those of WO_3_ and BiVO_4_ samples, confirming its best charge transfer capacity for water splitting [43]. Figure S3 shows the equivalent circuit of EIS spectra test and associated parameters [44]. From the parameters of equivalent circuit elements in the table, we can find that the *R*_ct_ of WO_3_/BiVO_4_ heterojunction is obviously less than that of original WO_3_ and BiVO_4_ photoanodes. It shows that heterojunction can effectively reduce the transfer resistance of photo-generated holes from electrode surface into electrolyte solution, which can be attributed to the formation of WO_3_/BiVO_4_ heterojunction with a favourable band position to accelerate the charge separation, and then the oxidation reaction of water on WO_3_ and BiVO_4_ photoanode surface is accelerated, and ultimately improve the photocatalytic performance [45]. The *J*–*V* curves of the photoelectrodes were obtained in phosphate buffer (0.5 M, pH = 7) including Na_2_SO_3_ (1 M) as hole scavenger under visible-light irradiation (Fig. 2c). The enlarged image of the potential (0.8–1.8 V vs. RHE) is shown in the inset of Fig. 2c. The photocurrent of the WO_3_/BiVO_4_ heterojunction electrode is higher than that for the individual WO_3_ and BiVO_4_ electrodes. In particular, the photocurrent of the WO_3_/BiVO_4_ sample attains 5.24 mA cm^−2^ at 1.23 V versus RHE, which is seven times more than that of the WO_3_ and twice more than that of the BiVO_4_, respectively. Notably, the current density under illumination is higher than that in the dark all the time from 0.6 to 1.6 V versus RHE, while the dark currents are almost zero in that region (Fig. S6). Furthermore, the photocurrent of WO_3_/BiVO_4_ electrodes can remain stable over 3 h (Fig. 2d). Even after 6 h, only a minimal loss in photocurrent density can be observed, indicating good photoelectrochemical stability (Fig. S7).

**Fig. 3:**
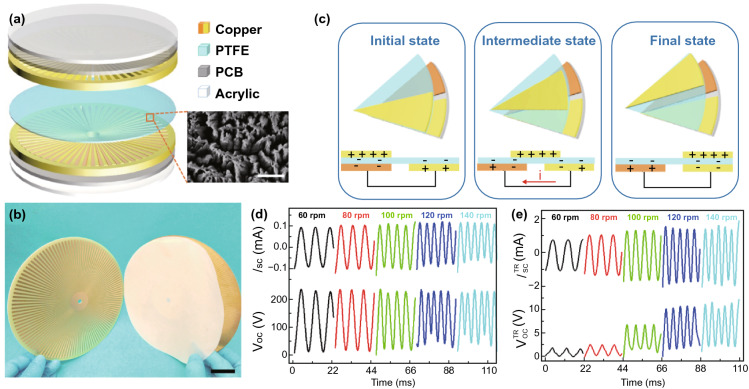
Schematic illustration, working mechanism and output performance of mechanical energy harvester. **a** Schematic diagram of RD-TENG. Inset: SEM image of PTFE surface with nanostructures (scale bar, 2 μm). **b** Photograph of the device model (scale bar, 2 cm). **c** Charge distributions scheme of the device. **d** Open-circuit voltage (*V*_oc_) and short-circuit current (*I*_sc_) before transformation and **e** after transformation between 60 and 140 rpm

The mechanical energy harvester, RD-TENG, is another important component of the self-powered PEC water-splitting system, as shown in Fig. 3. A RD-TENG with the multilayered structure consists of a rotator with disc shape and a matching stator (Fig. 3a, b). Two acrylic sheets were attached to the print circuit board (PCB) to serve as the supporting substrates. The copper film (central angle 1.5° and thickness 70 μm) works as a triboelectrification layer; meanwhile the PTFE film is used as the other triboelectrification layer. The SEM image shows the nanowires with a length of ~ 1 µm and a diameter of ~ 100 nm grown on the PTFE surface. The coupling effects of triboelectrification and electrostatic induction are the basic mechanism of RD-TENG (Fig. 3c) [46–49]. Due to different triboelectric polarities of the two triboelectrification layers, after a period of rotation, the copper surface and the PTFE surface will generate positive and negative electric charges, respectively. The initial state and the final state are deemed to be the states where the rotator corresponds to the left electrode and the right electrode. The process of the rotator spins from the initial state to the final state is defined as the intermediate state. Once the rotation starts, the surfaces of Cu and the PTFE will possess equal amount of negative and positive charges. After that, a potential difference will be produced between these two electrodes, and then a reverse current will be produced in the circuit until reaching the final state. As shown in Fig. 3d, the output characteristics of the RD-TENG under different speeds between 60 and 140 rpm have been measured. Under various rotation speeds, the value of *V*_oc_ is kept at ~ 230 V without obvious change, and *I*_sc_ increases as the speed raises, and the peak current reaches 0.12 mA at 140 rpm. After the transformation by a transformer, the output parameters of the RD-TENG, mainly *V*_oc_ and *I*_sc_, rise simultaneously with increasing the speed of rotation. The peak voltage and corresponding peak current increase to ~ 11 V and 1.6 mA, respectively, at the speed of 140 rpm (Fig. 3e).

**Fig. 4:**
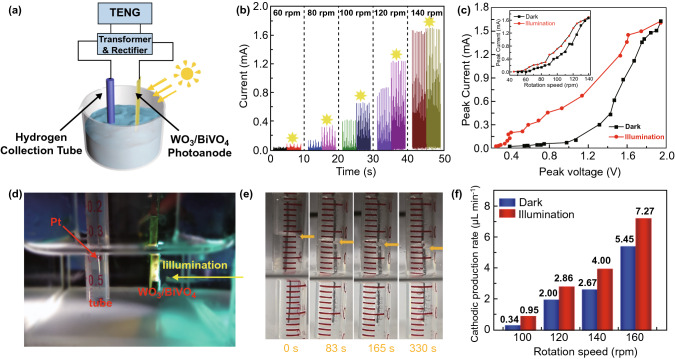
Demonstration of hybridized mechanical and solar energy-driven hydrogen production. **a** Schematic diagram of the self-powered photoelectrochemical hydrogen generation system. **b** Current at various rotation speeds under darkness or illumination. **c** Relationship between peak voltage and peak current in darkness or light. Inset: peak current under different rotation speeds, dark and illumination. **d** A photograph of the H_2_ collection tube was used to collect hydrogen under illumination. **e** Optical images of the H_2_ collection tube at the different times in a phosphate buffer (0.5 M, pH = 7) including Na_2_SO_3_ (1 M) as hole scavenger. **f** Hydrogen generation rate at four different rotation speeds in darkness or light

In order to illustrate the potential applications of the PEC hydrogen production system based on WO_3_/BiVO_4_ photoanode, we demonstrated a hybridized mechanical and solar energy-driven hydrogen production system, as shown in Fig. 4. The whole system consists of a RD-TENG, a transformer, a rectifier, the electrolytes, cathode and anode (Fig. 4a). Its equivalent circuit can be found in Fig. S4. As for electrolytic cell, a phosphate buffer (0.5 M, pH = 7) including Na_2_SO_3_ solution (1 M) as hole scavenger was selected as the electrolytes, a Pt electrode was used as the cathode, and the WO_3_/BiVO_4_ heterojunction photoanode was utilized as the anode. The Pt electrode was inserted into a H_2_ collection tube, where the H_2_ collection tube was fully filled with electrolytes, and then partly inserted into the electrolyser. According to the electrolysis effect, the PEC process occurred and hydrogen bubbles were produced at the cathode. Figure 4b demonstrates the trend of current of the self-powered hydrogen production system from 60 to 140 rpm under darkness or illumination. Obviously, the peak current has a significant increase after illumination. In addition, the peak photocurrent and the peak dark current exhibit the similar tendency with the increase in rotation speeds. However, the peak current sharply enhances to 0.1 mA at 60 rpm, while the current is almost zero in the dark. The generation of hydrogen will only take place under the light condition and hardly perform under darkness. When the rotating speed exceeds 130 rpm, the peak current is same in both illumination and dark conditions (see the illustration in Fig. 4c). Moreover, the peak voltage (equal to or greater than 1.61 V) is sufficient for directly electrolyzing water (Fig. S5) [50]; the peak current and peak voltage in darkness or under illumination were measured at various rotation speeds simultaneously (Fig. 4c). The peak current rises obviously along with the increase in the peak voltage, no matter in the light or in the dark condition. This result agrees with the trend of the *J*–*V* curve measured by the electrochemical workstation shown in Fig. 2c. To evaluate the H_2_ evolution characteristics of the self-powered PEC hydrogen generation system with electricity supplied by RD-TENG, a H_2_ collection tube was used to collect hydrogen, as shown in Fig. 4d. Figure 4e illustrates the optical images of the H_2_ collection tube varying with time when the rotating speed of the RD-TENG is 160 rpm. The volume of produced hydrogen gradually increases with increasing time, and an obvious dropping process of liquid level in the tube could be present. At the same time, distinct and continuous H_2_ bubbles were observed on the Pt electrode. The detailed dynamic water-splitting process can be intuitively seen in Supporting Movie S1. Furthermore, hydrogen evolution rate at four various speeds in darkness or under illumination is plotted in Fig. 4f. Particularly, at 160 rpm, the H_2_ generation rates are up to 5.45 μL min^−1^ under dark, and 7.27 μL min^−1^ under illumination, respectively. The corresponding energy conversion efficiency is calculated to be 2.43% and 2.59%, respectively (Supporting Note S3). Due to the peak output characteristics, the voltage output of RD-TENG does not always keep at the peak value. At these low voltages, PEC water splitting plays a leading role and the sunlight effect cannot be ignored. Thus, there are still significant differences between dark and illumination for the hydrogen generation rate.

## Conclusions

The WO_3_/BiVO_4_ heterojunction was prepared as a photoanode to generate H_2_ in a TENG-driven self-powered PEC water-splitting system. A RD-TENG furnished this system with external bias, and then simultaneously or separately converted mechanical energy and solar energy into hydrogen energy. When the rotation rate is 60 rpm, the peak photocurrent is 0.1 mA, the process of hydrogen production only happens under illumination conditions. When the rotation speed surpasses 130 rpm, the direct electrolysis of water is almost simultaneous with photoelectrocatalysis of water. The H_2_ production rates are quickly lifted to 5.45 and 7.27 μL min^−1^ at 160 rpm under dark and illumination, respectively. The corresponding energy conversion efficiency is calculated to be 2.43% and 2.59%, respectively. The heterojunction material is a benefit for the transfer and transmission of the photo-generated holes, thereby effectively lowering the composite of photo-generated electron–hole pairs. Understandably, the modification of photoanode material enables to boost the energy conversion efficiency in such a hybridized mechanical and solar energy-driven self-powered hydrogen production system.

## Electronic supplementary material

Below is the link to the electronic supplementary material.

Supplementary material 1 (PDF 826 kb)

Supplementary material 2 (AVI 4319 kb)
