# Supplementary material for: Hybridized Mechanical and Solar Energy-Driven Self-Powered Hydrogen Production
Source: Nanomicro Lett. 2020 Apr 9;12:88. doi: 10.1007/s40820-020-00422-4 (PMC7770943; doi:10.1007/s40820-020-00422-4)
Supplement: Supplementary file 1 — Supplementary material 1 (PDF 826 kb) [file 40820_2020_422_MOESM1_ESM.pdf]

Supporting Information for

Hybridized Mechanical and Solar Energies Driven Self-Powered Hydrogen Production

Xuelian Wei[1, 2], Zhen Wen[2, *], Yina Liu[3], Ningning Zhai[2], Aimin Wei[2], Kun Feng[2], Guotao Yuan[2], Jun Zhong[2], Yinghuai Qiang[1, *], Xuhui Sun[2, *]

[1]Jiangsu Province Engineering Laboratory of High Efficient Energy Storage Technology and Equipments, School of Materials Science and Engineering, China University of Mining and Technology, Xuzhou 221116, People's Republic of China

[2]Institute of Functional Nano and Soft Materials (FUNSOM), Jiangsu Key Laboratory for Carbon-Based Functional Materials and Devices, Soochow University, Suzhou 215123, People's Republic of China

[3]Department of Mathematical Sciences, Xi'an Jiaotong-Liverpool University, Suzhou 215123, People's Republic of China

*Corresponding authors. E-mail: wenzhen2011@suda.edu.cn (Z. Wen); yhqiang@cumt.edu.cn (Y. Qiang); xhsun@suda.edu.cn (X. Sun)

Supplementary Figures

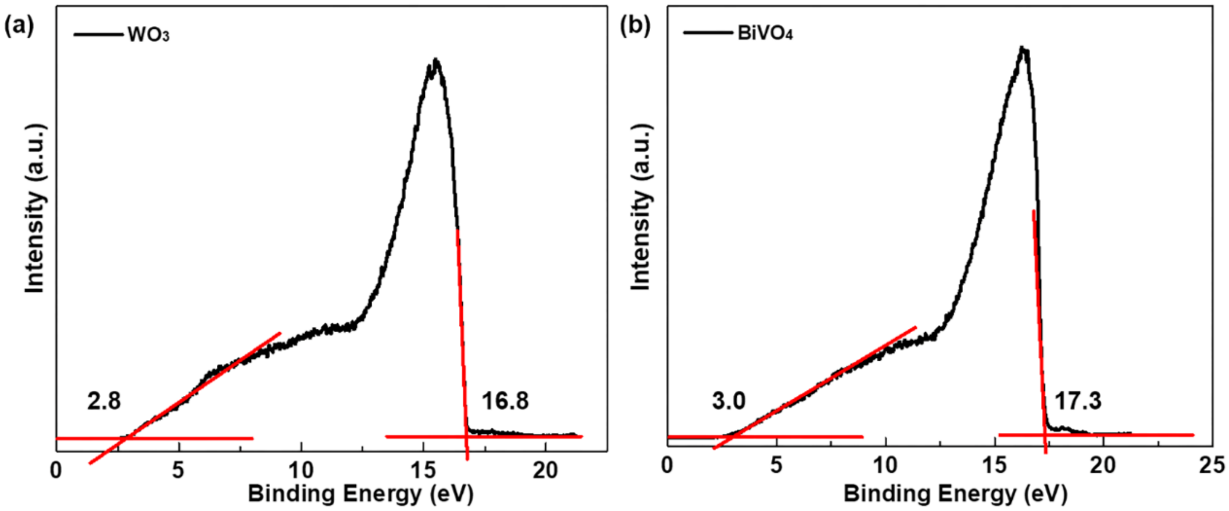

Fig. S1 UPS spectra of (**a**) WO_3 and (**b**) $BiVO_4$. The top of valence band is calculated to be about -7.2 eV and -6.9 eV (compared to the vacuum level) by subtracting the width of the He I UPS spectrum from the excitation energy (21.2 eV)

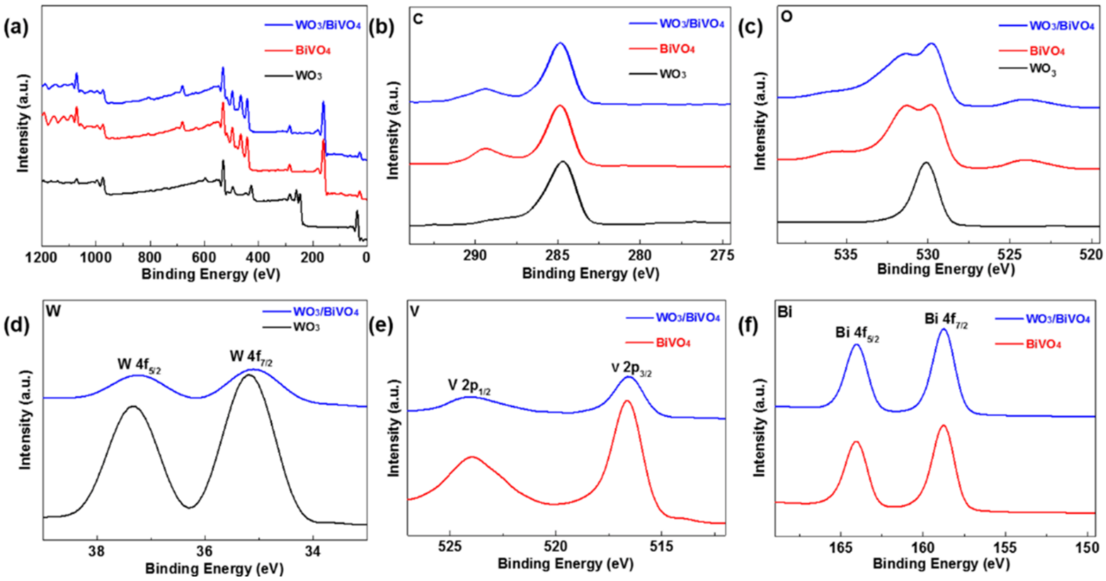

Fig. S2 XPS analysis of the WO_3, $BiVO_4$ and $WO_3/BiVO_4$ photoanodes: (**a**) Three pristine prepared photoanodes, (**b**) C, (**c**) O, (**d**) W 4f, (**e**) V 2p, and (**f**) Bi 4f spectra

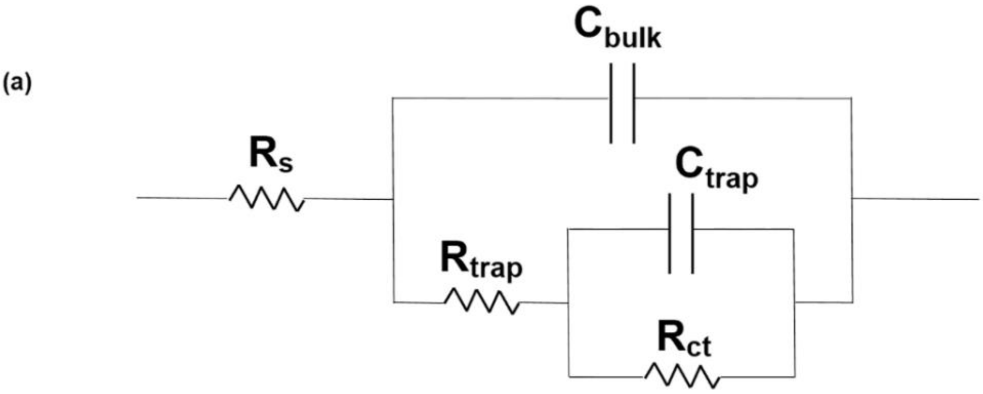

(b)

	R_s	C_{bulk} (10E-5)	R_{trap}	C_{trap} (10E-4)	R_{ct}
WO_3	0.01 ± 3E-4	0.77 ± 0.29	218.8 ± 15.32	0.98 ± 0.09	789.2 ± 23.41
$BiVO_4$	7.10 ± 0.06	10.52 ± 0.93	153 ± 28.38	4.89 ± 0.37	441.7 ± 37.54
$WO_3/BiVO_4$	5.28 ± 0.05	32.13 ± 5.57	26.44 ± 5.26	1.25 ± 0.42	355.7 ± 3.56

Fig. S3 (**a**) The equivalent circuit of EIS experimental test. (**b**) Parameters of equivalent circuit elements. R_s is the series resistances. C_{bulk} is the capacitance of the space charge depletion region at the electrode surface. R_{trap} is the resistance for trapping holes by surface states. C_{trap} shows the amount of active sites in surface states. R_{ct} is the resistance for charge transfer across the interface.

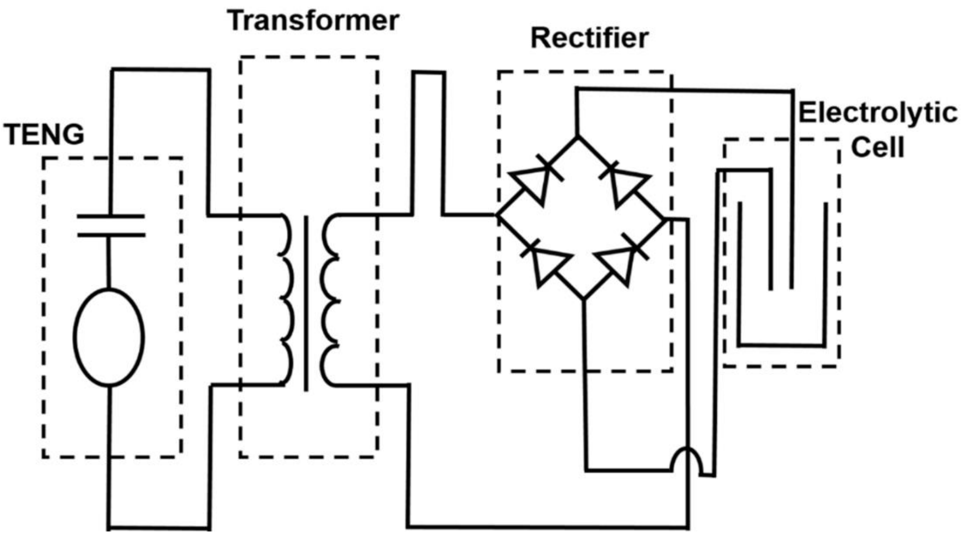

Fig. S4 Equivalent circuit of the self-powered solar hydrogen generation system

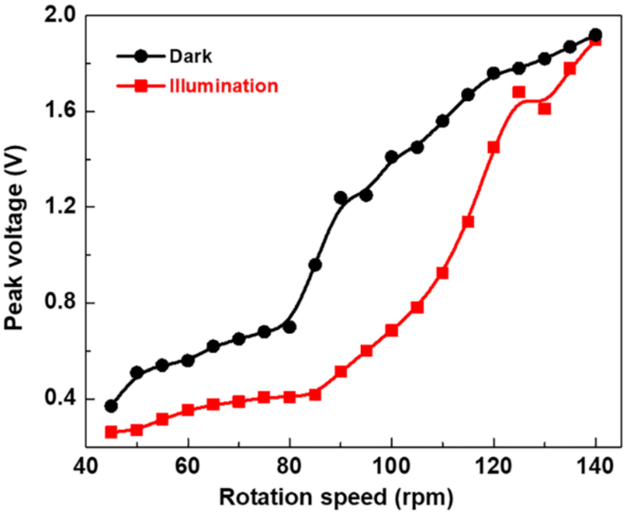

Fig. S5 Peak voltage as a function of different rotation speeds in the dark or under illumination

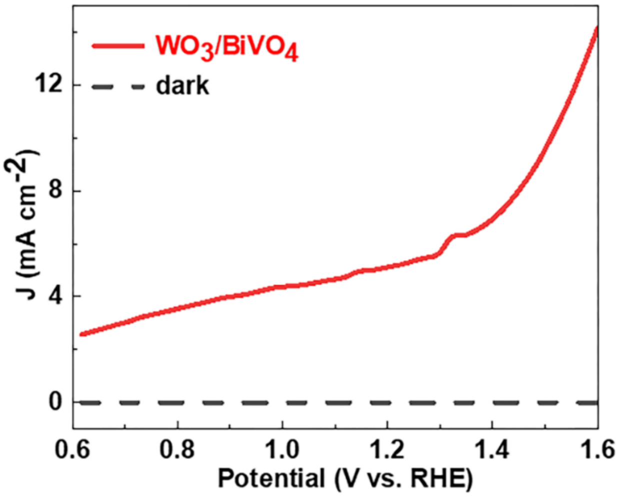

Fig. S6 *J-V* curves of $WO_3/BiVO_4$ electrodes measured in dark and under illumination

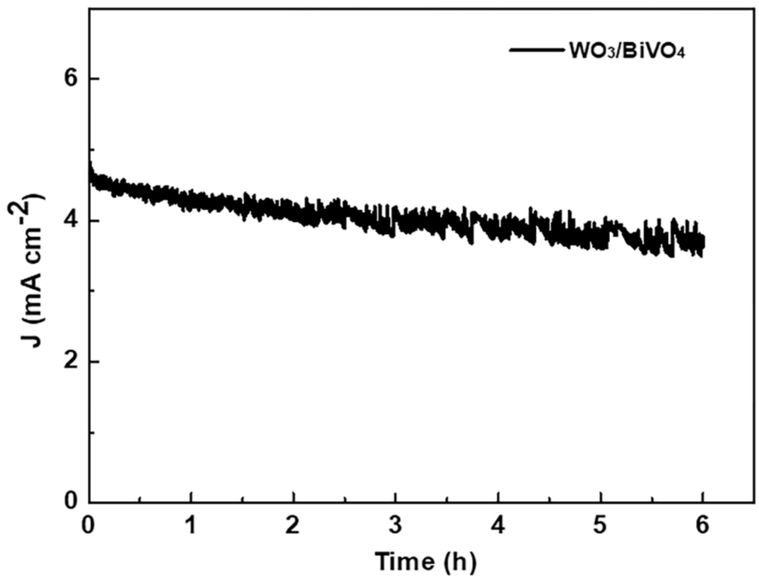

Fig. S7 Photochemical stability curve for the $WO_3/BiVO_4$ photoanode collected at 1.23 V vs. RHE over 6 h

Supporting Note S1

Figure S1 shows the UPS spectra of WO_3 and $BiVO_4$**.** E_{cutoff} is determined by linear extrapolation to zero of the yield of secondary electrons. From Fig. S1, E_{cutoff} = 16.8 eV for WO_3 and E_{cutoff} = 17.3 eV for $BiVO_4$. The HOMO energy is determined using the incident photon energy, hv = 21.2 eV, E_{cutoff}, and E_{onset}. Also, E_{onset} = 2.8 eV for WO_3 and E_{onset} = 3.0 eV for $BiVO_4$. Thus, the HOMO energies are obtained directly from the UPS measurements,

$$E_{HOMO} = hv - (E_{cutoff} - E_{onset}) \qquad (S1)$$

For WO_3, E_{HOMO} = -7.2 eV, and E_{HOMO} = -6.9 eV for $BiVO_4$. The LUMO energies were calculated using the HOMO levels and the optical gaps (E_g) obtained from the onset of absorption (Fig. 1f). E_g = 2.58 eV for WO_3 and E_g = 2.41 eV for $BiVO_4$. Thus for WO_3, E_{LUMO} = -4.62 eV; and E_{LUMO} = -4.49 eV for $BiVO_4$.

Supporting Note S2

The slopes from the Mott-Schottky plots are used to estimate the carrier densities using Eq. S2:

$$N_d = (2/e_0\varepsilon\varepsilon_0)[d(1/C^2)/dV]^{-1} \qquad (S2)$$

where e_0 is the electron charge (1.602×10^{-19} C), ε is the dielectric constant of WO_3 (2.3), $BiVO_4$ (60), ε_0 is the permittivity of vacuum (8.854×10^{-12} F m^{-1}), N_d is the donor density and V is the potential applied at the electrode. Capacitances were derived from the electrochemical impedance obtained at each potential with 10,000 Hz frequency in the dark. The Mott-Schottky slopes of both samples are positive which indicates that they are n-type semiconductors with electrons as majority carriers. The slopes are used to estimate carrier densities. The charge carrier densities after calculation for WO_3 and $BiVO_4$ are 6.81×10^{23} and 7.30×10^{19} cm^{-3}, respectively. Moreover, $BiVO_4$ can only be considered as a modification in our experiment and very small quantity, so the result of the $WO_3/BiVO_4$ heterojunction

can be supposed to be as the WO_3, while the value is 6.81×10^{23} cm^{-3}. The higher carrier density of the $WO_3/BiVO_4$ heterojunction photoanode compared with the individual sample $BiVO_4$ could partly provide a benefit to the performance.

Supporting Note S3

$$\text{Overall energy conversion efficiency } (\eta) = \frac{\text{Output Energy}}{\text{Total Input Energy}} = \frac{E_1}{E_2 + E_3}$$

where E_1 is the energy of produced hydrogen involved, E_2 is the input mechanical energy to drive a RD-TENG, and E_3 is the input sunlight light energy.

When the rotation speed is 160 rpm, the calculation process of different energies is as follows:

(1) Under illumination, the H_2 production rates reached 7.27 μL/min, thus,

$$E_1 = n_1 \Delta G = \frac{7.27 \times 10^{-6}}{22.4} \times 237 \times 10^3 = 7.69 \times 10^{-2}\ J$$

Under dark, the H_2 production rates reached 5.45 μL/min, thus

$$E_1' = n_1' \Delta G = \frac{5.45 \times 10^{-6}}{22.4} \times 237 \times 10^3 = 5.77 \times 10^{-2}\ J$$

where n_1 and n'_1 is moles of hydrogen under illumination and dark, , ΔG is standard Gibbs free energy.

(2) The rated torque of the rotary motor to drive the RD-TENG is 8.5 N m, thus,

$$E_2 = \frac{T \cdot n}{9550} \cdot t = \frac{8.5 \times 160}{9550} \times 1000 \times \frac{1}{3600} \times 60s = 2.37\ J$$

where T is the torque of the rotary motor, n is the rotation speed, t is the time.

(3) All illuminated area is 0.1 cm^2, thus,

$$E_3 = P \cdot S \cdot t = 1000 W/m^2 \times (0.1 \times 10^{-4})\ m^2 \times 60s = 0.6\ J$$

where P is the optical power density, S is the illuminated area, t is the time.

In conclusion,

$$\eta = \frac{E_1}{E_2 + E_3} \times 100\% = 2.59\%$$

$$\eta' = \frac{E_1'}{E_2} \times 100\% = 2.43\%$$
